# Effects of Chitosan–Gentamicin Conjugate Supplement on Non-Specific Immunity, Aquaculture Water, Intestinal Histology and Microbiota of Pacific White Shrimp (*Litopenaeus vannamei*)

**DOI:** 10.3390/md18080419

**Published:** 2020-08-10

**Authors:** Fengyan Liang, Chengpeng Li, Tingting Hou, Chongqing Wen, Songzhi Kong, Dong Ma, Chengbo Sun, Sidong Li

**Affiliations:** 1 School of Chemistry and Environmental Science, Guangdong Ocean University, Zhanjiang 524088, China; yaner204126@163.com (F.L.); htt0415@126.com (T.H.); kongsongzhi@126.com (S.K.); 13702737491@163.com (S.L.); 2 Department of Fisheries, Guangdong Ocean University, Zhanjiang 524088, China; chongqingwen@163.com; 3 Department of Biomedical Engineering, Jinan University, Guangzhou 510632, China; tmadong@jnu.edu.cn; 4 Southern Marine Science and Engineering Guangdong Laboratory, Zhanjiang 524025, China

**Keywords:** *Litopenaeus vannamei*, chitosan–gentamicin conjugate, nonspecific immunity parameters, intestinal morphology, 16S rRNA gene sequencing, microbiota

## Abstract

When the aquaculture water environment deteriorates or the temperature rises, shrimp are susceptible to viral or bacterial infections, causing a large number of deaths. This study comprehensively evaluated the effects of the oral administration of a chitosan–gentamicin conjugate (CS-GT) after *Litopenaeus vannamei* were infected with *Vibrio parahaemolyticus,* through nonspecific immunity parameter detection, intestinal morphology observation, and the assessment of microbial flora diversification by 16S rRNA gene sequencing. The results showed that the oral administration of CS-GT significantly increased total hemocyte counts and reduced hemocyte apoptosis in shrimp (*p* < 0.05). The parameters (including superoxide dismutase, glutathione peroxidase, glutathione, lysozyme, acid phosphatase, alkaline phosphatase, and phenoloxidase) were significantly increased (*p* < 0.05). The integrity of the intestinal epithelial cells and basement membrane were enhanced, which correspondingly alleviated intestinal injury. In terms of the microbiome, the abundances of *Vibrio* (Gram-negative bacteria and food-borne pathogens) in the water and gut were significantly reduced. The canonical correspondence analysis (CCA) showed that the abundances of *Vibrio* both in the water and gut were negatively correlated with CS-GT dosage. In conclusion, the oral administration of CS-GT can improve the immunity of shrimp against pathogenic bacteria and significantly reduce the relative abundances of *Vibrio* in aquaculture water and the gut of *Litopenaeus vannamei*.

## 1. Introduction

Shrimp farming is one of the most important industries in the world [1,2]. Due to high yield, fast growth, and easy adaptability, Pacific white shrimp (*Litopenaeus vannamei*) have become one of the most profitable species in shrimp farming, with an annual global production of more than 4.1 million tons [3]. This accounts for 73% in China [4]. The Pacific white shrimp belongs to temperate and tropical shrimp. It can survive in the temperature of 10–40 °C, and the most suitable temperature range is 25–32 °C. It can grow within a salinity range of 28–34%_0._ Like other shrimps, Pacific white shrimp lack adaptive immune mechanisms and depend on innate immune responses for the detection and elimination of pathogens. However, it is estimated that about 1.7 million tonnes of shrimp has been lost in recent years due to the environmental deterioration of aquaculture systems [4]. Among them, *Vibrios* (including *Vibrio parahaemolyticus*, *Vibrio harveyi* and *Vibrio alginolyticus*) are responsible for causing diseases in shrimps and human gastroenteritis disease after the consumption of contaminated seafood [5,6]. When the aquaculture water environment deteriorates or the temperature rises (especially in summer), the *Vibrio* outbreaks result in significant shrimp deaths. To prevent or control *Vibrio*, a range of strategies are used; for example, antibiotics have been used as the main treatment for *Vibrio*. However, the excessive or inappropriate use of antibiotics may increase the prevalence of multi-drug-resistant strains, environmental pollution, and residue accumulation. In response to these concerns, alternative strategies have been proposed, including plant extracts [7,8], probiotics [4,9], polysaccharides [10,11], and other feed supplements [12,13]. However, these strategies are ineffective; once the *Vibrio* break out, the shrimps will still die in large numbers.

Chitosan (CS), a cationic polysaccharide, is obtained from the N-deacetylation of chitin [14]. Due to its potential in immune modulation, antioxidant function, biocompatibility, and wide-spectrum antibacterial activity [15], chitosan has been widely applied in aquaculture [16,17,18,19]. For example, one study showed that the performance and health of Nile tilapia (*Oreochromis niloticus*) were significantly improved when chitosan nanoparticles were loaded in basic diets (one gram per kilogram) [20]. Similarly, Khani Oushani et al. found that diets supplemented with 0.05 g kg^−1^ of nano-chitosan and clinoptilolite could improve the growth performance and immune parameters of rainbow trout (*Oncorhynchus mykiss*) [21,22,23,24]. However, these studies mentioned above principally aimed to improve the immunity of aquatic animals. However, there are few reports relating to the application of chitosan on aquatic pathogenic bacteria, for the antibacterial activity of chitosan is reported to be poor. Recently, a series of chitosan derivatives with improved antibacterial activity have been reported [25]. Gentamicin (GT) has abundant amine groups and high water solubility, and can penetrate the bacterial cell membrane and inhibit the synthesis of bacterial proteins. In 2019, we successfully synthesized a chitosan–gentamicin conjugate (CS-GT) via oxidation and a Schiff base reaction. Compared to CS, its water-solubility and antimicrobial activity are significantly improved [26].

Our previous research also found that CS-GT-based hydrogels, with antimicrobial activity, cytocompatibility, and hemocompatibility, can accelerate the wound healing of New Zealand rabbits [27]. Particularly, CS-GT can inhibit pathogenic *Escherichia coli*, *Pseudomonas aeruginosa*, and *Staphylococcus aureus* effectively [26]. Therefore, CS-GT may be potentially applied in aquatic farming. The aim of present study was to understand the effects of CS-GT on immunity, intestinal histology, and in vitro and in vivo microbiota, as well as their functions in *Litopenaeus vannamei* after infection with *Vibrio parahaemolyticus*. These results are expected to better clarify the mechanisms involved in the oral administration of CS-GT for *Litopenaeus vannamei* farming and provide useful guidance for chitosan derivative development in aquaculture. 

## 2. Results and Discussion

### 2.1. Water Physicochemical Quality

**Figure 1:**
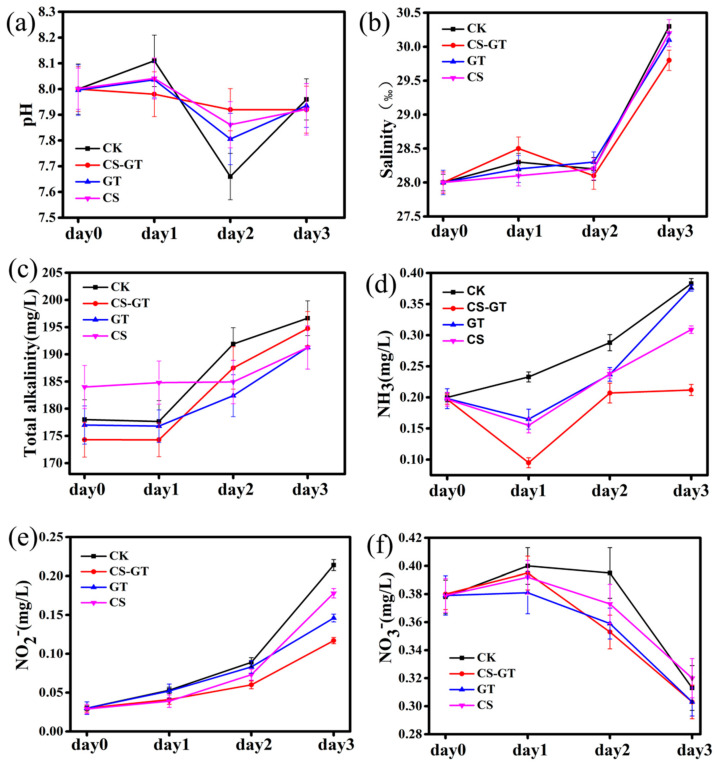
Water physical–chemical constituents: (**a**) pH, (**b**) salinity, (**c**) total alkalinity, (**d**) NH_3_ (mg/L), (**e**) NO_2_^−^ (mg/L), and (**f**) NO_3_^−^ (mg/L) (mean ± SD, n = 3).

Water quality monitoring is important in aquaculture. The physical–chemical constituents of the water often directly promote the growth of *Litopenaeus vannamei*, and ammonia nitrogen, nitrite nitrogen, and nitrate nitrogen are important standards for monitoring water quality. The pH, salinity, total alkalinity, ammonia nitrogen (NH_3_), nitrate nitrogen (NO_3_^−^) and nitrite nitrogen (NO_2_^−^) indexes in the CK (infected with *Vibrio parahaemolyticus*), CS-GT (supplemented with CS-GT after infection with *Vibrio parahaemolyticus*), GT (supplemented with GT after infection with *Vibrio parahaemolyticus*), and CS (supplemented with CS after infection with *Vibrio parahaemolyticus*) groups were measured every day during the experiment. As shown in Figure 1, the pH values of all groups were in the range of 7.66 to 8.11. The salinity, total alkalinity, NH_3_, and NO_2_^−^ in all the groups were increased, while the NO_3_^−^ content in all groups was decreased. The overall change trends of NH_3_ and NO_2_^−^ were similar to those in studies by other experts [28,29]. It is likely that the excessive input of feeds and massive deposition of fecal matter during the feeding period—causing organic matter accumulation, decomposition, and oxygen consumption, resulting in the bottom of tank entering a hypoxic state, a large number of anaerobic bacteria multiplying, and the incomplete decomposition of organic matter—produced a large number of toxic and harmful physical–chemical factors such as ammonia nitrogen and nitrous nitrogen [30]. It is worth noting that the concentrations of NH_3_ and NO_2_^−^ in the CS-GT group were the lowest compared to other groups.

### 2.2. Hematological Profile

**Figure 2:**
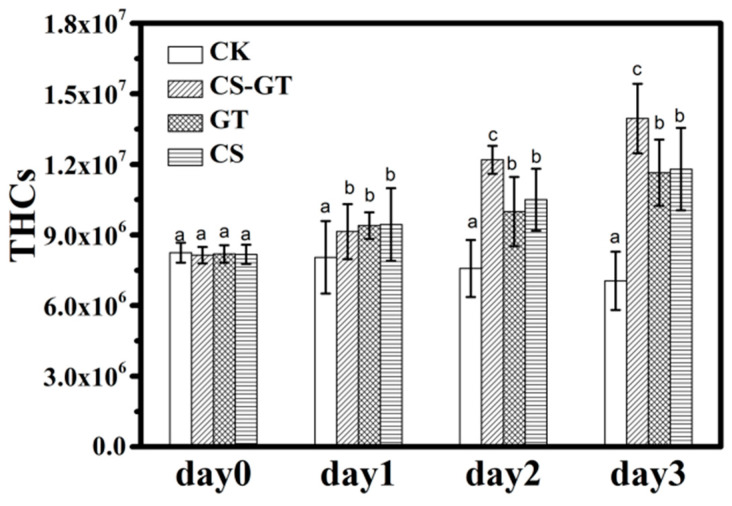
The total hemocyte counts (THCs) of the shrimp in each group (mean ± SD, n = 6) (different letters indicate significant differences between the different groups at the same time point (*p* < 0.05)).

**Figure 3:**
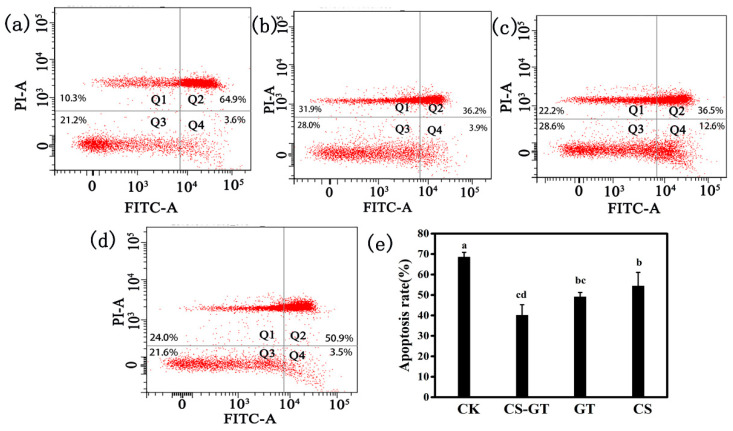
Flow cytometry detection of hemocyte apoptosis in (**a**) CK, (**b**) CS-GT, (**c**) GT, and (**d**) CS groups, and (**e**) hemocyte apoptosis rate (mean ± SD, n = 6). CK: Infected with *Vibrio parahaemolyticus* group; CS-GT: Supplemented with CS-GT after infection with *Vibrio parahaemolyticus* group; GT: Supplemented with GT after infection with *Vibrio parahaemolyticus* group; CS: Supplemented with CS after infection with *Vibrio parahaemolyticus* group. (different letters indicate significant differences between the different groups at the same time point (*p* < 0.05)).

During the experiment, the mortality rates were about 12%, 9%, 7%, and 5% in the CK group, CS group, CS-GT group, and GT group, respectively. The hemocytes related to humoral immunity, play a crucial part in immune defense system. Therefore, hemocytes can be used to reflect the immune response of the shrimps, and they have been reported to decrease the exposure of shrimps to infectious pathogens or environmental stress, thereby reducing the risk of secondary infections [31,32]. In this study, the total hemocyte counts (THCs) of each group were counted each day using an inverted phase-contrast microscope. As shown in Figure 2, the THCs of the supplement groups significantly increased compared to those in the CK group (*p* < 0.05). The THCs of CS-GT group were significantly higher than those of the GT and CS groups (*p* < 0.05). This may be due to the supplements, especially CS-GT, which increased THCs through the proliferation of hemocytes and enhancement of their phagocytic activity. The result was similar to that of Zhai et al., who found that enrofloxacin (ENR) and San-Huang-San (SHS) promoted disease resistance in shrimp [32]. The hemocyte apoptosis assays were conducted with an Annexin V-FITC/PI Apoptosis Detection Kit at Day 3 according to the manufacturer’s protocol. As shown in Figure 3a–d, the CK group showed the most hemocyte apoptosis, while the CS-GT and GT groups showed the least. As shown in Figure 3e, the hemocyte apoptosis rate of the CK group was the highest, which was significantly higher than that of the supplement groups (*p* < 0.05). There was no significant difference in apoptosis rate between the supplement groups (*p* > 0.05). The results indicate that the supplementation can effectively reduce hemocyte apoptosis in *Litopenaeus vannamei* infected with *Vibrio parahaemolyticus*. 

### 2.3. Nonspecific Immunity Parameters

**Figure 4:**
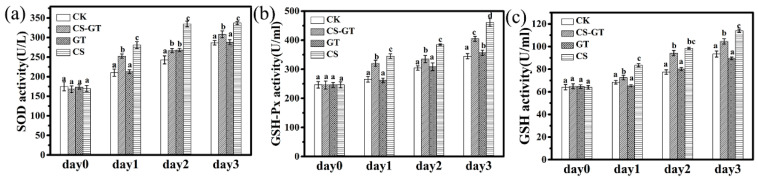
The antioxidant activities of shrimp in each group (mean ± SD, n = 6). (**a**) Superoxide dismutase (SOD), (**b**) glutathione peroxidase (GSH-Px), and (**c**) glutathione (GSH). (different letters indicate significant differences between the different groups at the same time point (*p* < 0.05)).

Antioxidant activities are indicators of the antioxidant status and oxidative stress of aquatic animals. Previous research has showed that, once reactive oxygen species (ROS) production increases, organisms are able to activate a series of antioxidant defense systems such as superoxide dismutase (SOD), catalase (CAT), and the glutathione triad—glutathione (GSH), glutathione s-transferase (GST), and glutathione peroxidase (GSH-Px)—to detoxify the ROS, preventing or repairing oxidative damage [33]. SOD is a molecular biomarker for evaluating the oxidative stress status of aquatic organisms, due to it catalyzing the dismutation of superoxide anions to hydrogen peroxide and molecular oxygen, forming a first-line antioxidant enzymatic defense system [34]. GSH-Px can remove lipid peroxides induced by reactive oxygen species and •OH, protecting the integrity of cell membrane structure and function. GSH works with glutathione peroxidase to reduce hydrogen peroxide to water, thereby maintaining the integrity of red blood cell membranes and protecting red blood cells from damage by oxidants [35]. In this study, the SOD, GSH-Px, and GSH activities of each group were measured each day using commercial detection kits. As shown in Figure 4a–c, the SOD, GSH-PX, and GSH activities in the CK group increased after infection from Day 1 to Day 3; the results were similar to those of the previous study, which showed that the SOD, GSH-Px, and GSH activities of *Litopenaeus vannamei* increased after infection compared to those in the control group (healthy shrimp) [36]. This may be due to the fact that the shrimp can produce a natural immune response and activate the antioxidant system, which play an important role in clearing the excess ROS [36]. In the supplement groups, the SOD, GSH-Px, and GSH activities in CS-GT and CS groups were significantly higher than those in CK and GT groups (*p* < 0.05). The antioxidant activities in the CS group were the highest compared to those in the other groups. This may be due to the fact that the elevated levels of SOD, GSH, and GSH-Px activities upon the oral administration of CS-GT are related to a greater reduction in the production of ROS and lipid peroxidation in *Litopenaeus vannamei*.

**Figure 5:**
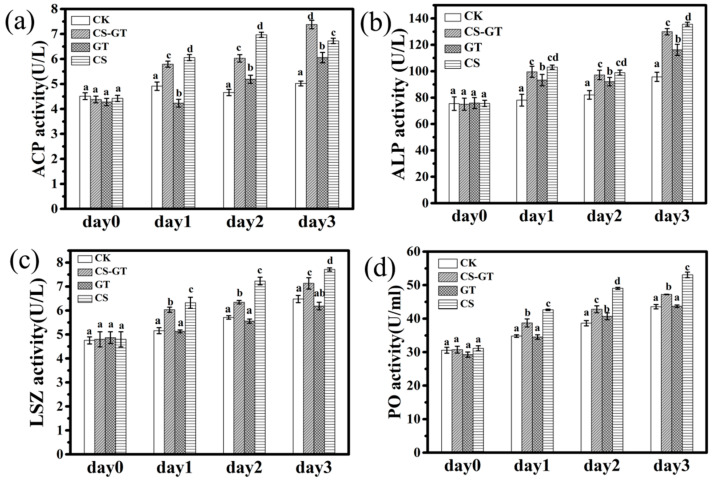
The immune enzyme activities of shrimp in each group (mean ± SD, n = 6). (**a**) Acid phosphatase (ACP), (**b**) alkaline phosphatase (ALP), (**c**) lysozyme (LSZ), and (**d**) phenoloxidase (PO). (different letters indicate significant differences between the different groups at the same time point (*p* < 0.05)).

Previous research demonstrated that various immune enzymes including phenoloxidase (PO), acid phosphatase (ACP), alkaline phosphatase (ALP), and lysozyme (LSZ) are generally selected as an indicator to evaluate the immune status and disease resistance of shrimp [10,11]. ACP and ALP are composed of many kinds of phosphomonoesterases, which are very important to the crustacean immune system [37]. Furthermore, ACP is an important component of phagocytic lysosomes, and in the phagocytosis and encapsulation of hemocytes, phagocytic lysosomes play a bactericidal role with the release of ACP [32,38]. ALP is a kind of phosphomonoesterase that aids in detoxification during normal living and phagolysis and in the digestion and absorption of many nutrients [39]. Lysozyme (LSZ) is a component of the innate immune system of invertebrates, functioning as an antibacterial protein [40]. It hydrolyzes mucopolysaccharides, which are basic components of the bacterial cell wall and kill pathogens [41]. In many invertebrates, the PO cascade represents a critical host defense response. PO is the terminal enzyme in the pro-PO activation system and involved in host defense reactions such as wound healing, cytotoxicity, and phagocytosis [11,42]. In this study, the immune enzyme activities of each group were measured each day using commercial detection kits. As shown in Figure 5, the ALP, PO, and LSZ activities in the CK group increased from Day 1 to Day 3, while the ACP activity peaked at Day 1 then decreased from Day 2 to Day 3 but was still higher than at Day 0. The results were similar to those in the previous study, which showed that the ACP, ALP, and LSM activities of *Litopenaeus vannamei* increased after infection compared to those in the control group (healthy shrimp) [32]. This might because of *Vibrio parahaemolyticus* infection leading to a stress reaction in the shrimp, inducing an immune response [32]. In the supplement groups, the ACP activity in the CS-GT and CS groups was significantly higher than that in the CK and GT groups (*p* < 0.05) (Figure 5a); it was highest in the CS-GT group at Day 3 (*p* < 0.05). The ALP activity in the supplement groups was significantly higher than that in CK group (*p* < 0.05) (Figure 5b). The LSZ and PO activities in the CS-GT and CS groups were significantly higher than those in the CK and GT groups (*p* < 0.05) (Figure 5c,d). In conclusion, immune enzyme (PO, LSZ, ACP, and ALP) activities in the CS-GT group were significantly higher than those in the CK group in this study. This may be the reason that the oral administration of CS-GT further enhanced the immune response of *Litopenaeus vannamei* to eliminate or kill the pathogens once they invaded [32].

### 2.4. Intestinal Histology

**Figure 6:**
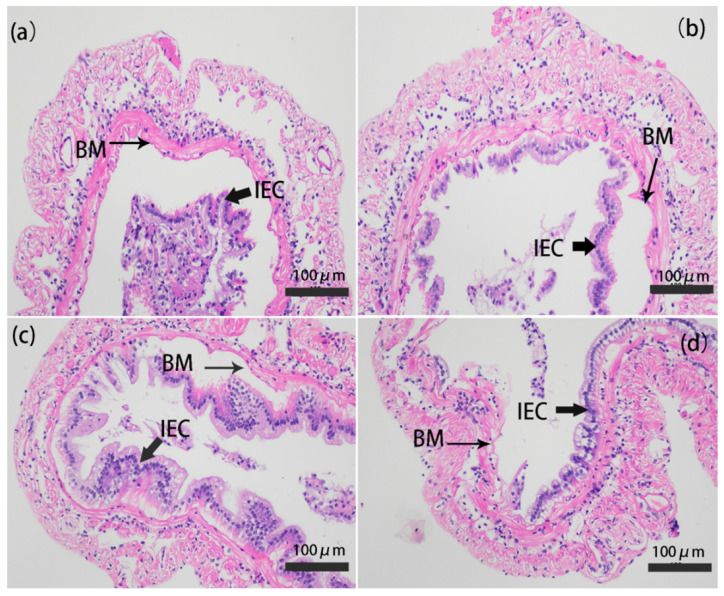
Photomicrographs of gut cross-cutting in (**a**) CK, (**b**) CS-GT, (**c**) GT, and (**d**) CS groups (mean ± SD, n = 3). The magnification was 200×, and the scale bar represents 100 µm. CK: Infected with *Vibrio parahaemolyticus* group; CS-GT: Supplemented with CS-GT after infection with *Vibrio parahaemolyticus* group; GT: Supplemented with GT after infection with *Vibrio parahaemolyticus* group; CS: Supplemented with CS after infection with *Vibrio parahaemolyticus* group; IEC: Intestinal epithelial cells; BM: Basement membrane.

The intestine in vertebrates plays a critical role in metabolism, nutrient absorption, and immune function [43]. In the present study, the intestinal histology of shrimp at Day 3 was observed by staining with hematoxylin and eosin (H&E). The microstructure of the intestine in only infected group was lesions (Figure 6a); epithelial cells were completely detached from the basement membrane and severely destroyed. Parts of epithelial cells were detached from the basement membrane and destroyed in the CS-GT and GT groups (Figure 6b,c). In most regions of the intestine in the CS group, epithelial cells were detached from the basement membrane and severely destroyed (Figure 6d). The results indicated that the oral administration of CS-GT and GT could enhance the integrity of the intestinal epithelial cells and basement membrane, which correspondingly alleviated intestinal injury. This may be due to the antibacterial effect of CS-GT and GT. The results were similar to those in the previous studies, which have shown that dandelion extract can improve the intestinal structure and intestinal immune function of the juvenile golden pompano (*Trachinotus ovatus*) [44].

### 2.5. Microbiota

#### 2.5.1. Alpha Diversity Analysis

**Figure 7:**
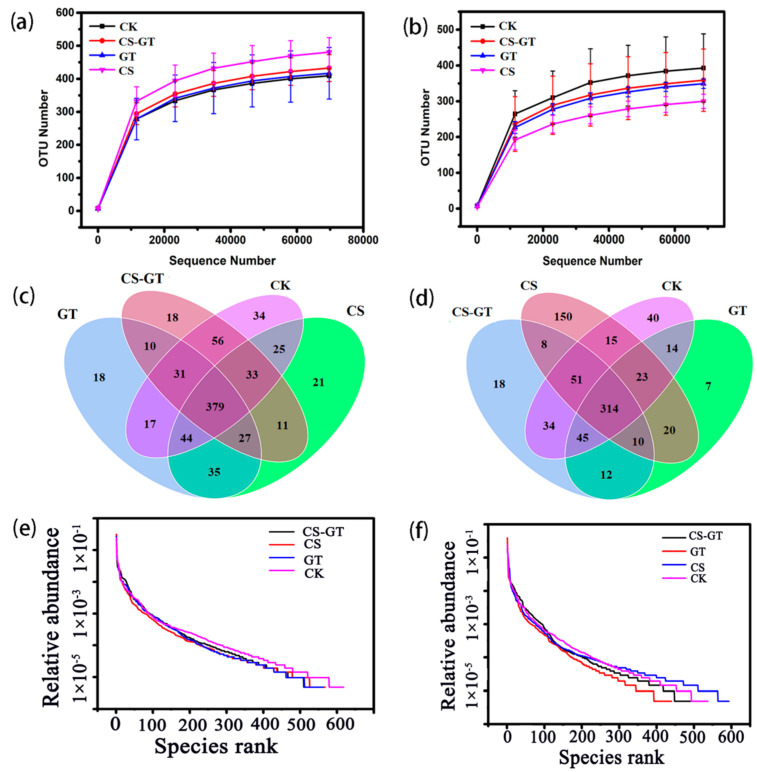
Rarefaction curves of water (**a**) and gut (**b**) samples; Venn diagram shows the operational taxonomic unit (OTU) numbers of water (**c**) and gut (**d**) samples; rank abundance curve of water (**e**) and gut (**f**) samples (mean ± SD, n = 3).

A total of 12 water samples and 12 intestinal samples (n = 3) were collected for 16S rRNA gene sequencing, respectively. After data quality filtering, an average of 80,150 and 80,104 high-quality reads were obtained from water and gut samples, respectively. For all the water and gut samples, their rarefaction curves (Figure 7a,b) gradually approach the saturation plateau, showing reliable sequencing and sufficient diversity. A Venn diagram of four independent ovals is presented in Figure 7c,d, which are assigned to the four experimental groups, respectively. As shown in Figure 7c, for the water samples, the operational taxonomic units (OTUs) in the CK group (i.e., 619) are higher than those in the supplementation groups. On the contrary, for the gut samples, the OTUs in the CK group (i.e., 536) are lower than those in the CS group (i.e., 591) (Figure 7d) but still higher than those in the CS-GT (i.e., 492) and GT (i.e., 445) groups. According to the rank abundance curve (Figure 7e,f), the microbial species richness of all the groups with dietary supplementation was decreased compared to that in the CK group, except for the gut sample in the CS group.

After dietary supplementation, the Chao1 (total species richness), ACE (abundance-based coverage estimator), and Shannon indices of alpha diversity were all calculated and are shown in Table 1. Conversely, the Simpson indices of alpha diversity were all improved. Generally speaking, there are no significant differences among the four groups for both the water and gut samples, except for the Shannon index of the GT group and Simpson indices of the CS-GT and GT groups in the water samples. In addition, all the Good’s coverage indices are approaching 1, indicating that the OTUs obtained from each library can represent the majority of bacteria in the water and gut.

#### 2.5.2. Beta Diversity Analysis

**Figure 8:**
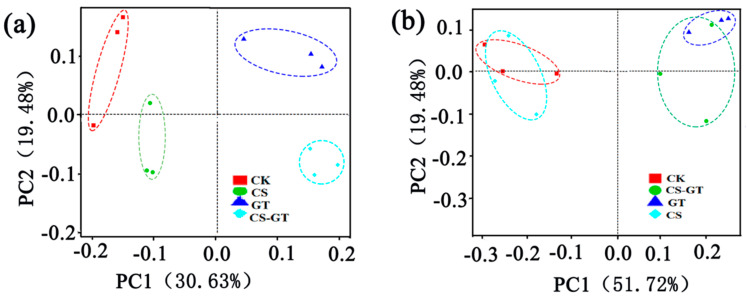
Principal coordinates analysis (PCoA) plot based on weighted-UniFrac analysis of water (**a**) and guts (**b**) of shrimp (n = 3). The abscissa represents one principal component, the ordinate represents another principal component, and the percentage represents the contribution of the principal component to the sample difference.

The beta diversity was used to analyze the differences between the microbial communities in the water and gut samples. Principal coordinates analysis (PCoA) based on the weighted-uniFrac distance for bacterial profiles was used in the present study to indicate the community changes in different samples. The PCoA showed that, both in the water and intestinal samples, the CS-GT and GT groups were clustered on the right side of the abscissa, and the distance was closer (Figure 8a); the CK and CS groups were clustered on the left side of the abscissa, and the distance was closer. The results showed that the microbial communities of the CS-GT and GT groups were similar, as were those of the CK and CS groups (Figure 8b). It may be related to the fact that both CS-GT and GT can inhibit Gram-negative bacteria.

#### 2.5.3. Microbial Composition and Changes

**Figure 9:**
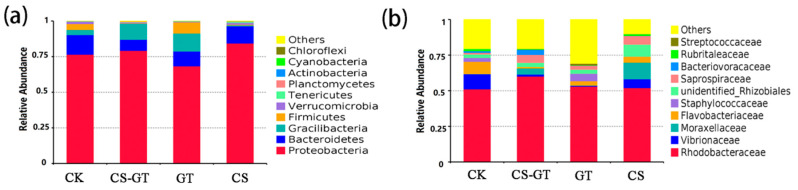
Composition and relative abundance of water microbial communities (**a**) at phylum level and (**b)** at family level (mean ± SD, n = 3).

**Figure 10:**
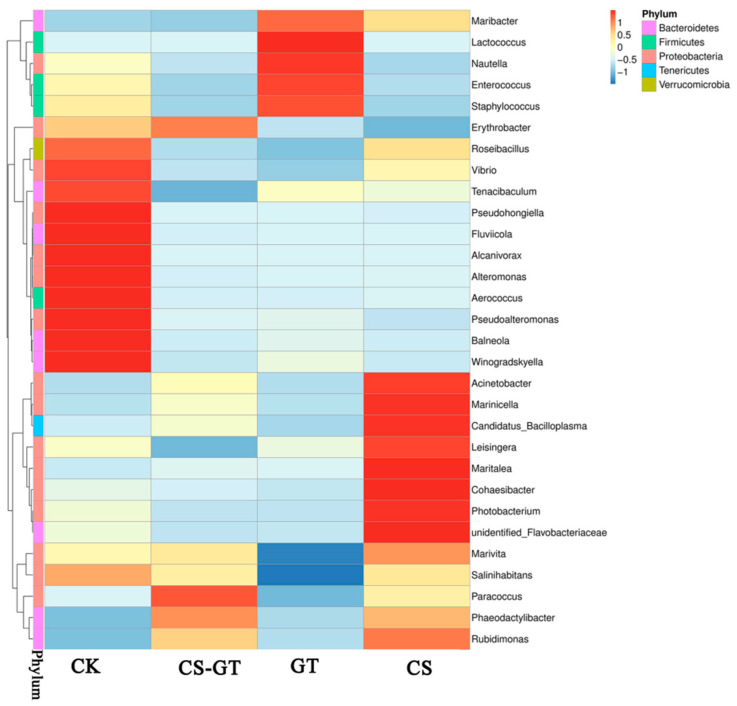
Heatmap analysis of the species abundance clustering in the top 30 at the genus level in the water (mean ± SD, n = 3).

Bacteria present in the aquaculture environment play an important role in the processes of nutrient cycling and mineralization of organic compounds. The microbial composition and changes in the water in each group were analyzed. At the phylum level (Figure 9a), the bacteria were predominantly Proteobacteria (62.28–92.22%), Bacteroidetes (11.23–22.56%), Gracilibacteria (5.01–22.35%), Firmicutes (0.25–11.13%), Verrucomicrobia (0.32–1.13%), and Tenericutes (0.14–1.08%). The results showed that Proteobacteria (62.28–92.22%) was the most abundant phylum in all the groups, which was consistent with previous studies [45,46,47], likely implying that Proteobacteria comprised the largest and most phenotypically diverse division of the prokaryotes [48]. Bacteroidetes was another predominant phylum; the higher abundance of Bacteroidetes might be caused by pollutants such as nitrates, ammonia, and feces [49]. At the family level (Figure 9b), Rhodobacteraceae (53.90–71.49%) was the most abundant in all the groups, followed by Vibrionaceae (15.38–29.90%), Moraxellaceae (8.76–10.21%), Flavobacteriaceae (5.75–6.86%), and Staphylococcaceae (0.58–1.01%). Rhodobacteraceae showed the highest abundance in all the groups, especially in the CS-GT group. Rhodobacteraceae, as a specific taxon in aquaculture water, is associated with algae. Some members of Rhodobacteraceae can produce tropodithietic acid (TDA) to inhibit the growth of pathogens [46]. Especially, *Vibrio* have been regarded as opportunistic pathogens causing stress and disease infections [50]. The abundances of *Vibrio* were much lower in the GT and CS-GT groups than in the CK group at the genus level (Figure 10). This may be due to the strong bactericidal effect of CS-GT and GT on Gram-negative bacteria. 

**Figure 11:**
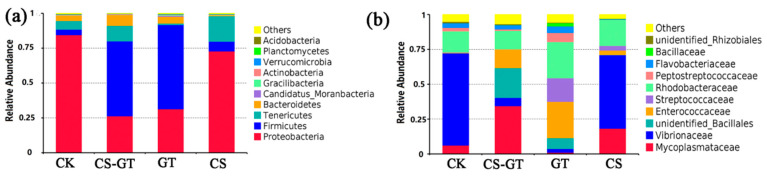
Composition and relative abundance of gut microbial communities (**a**) at phylum level and (**b**) at family level (mean ± SD, n = 3).

**Figure 12:**
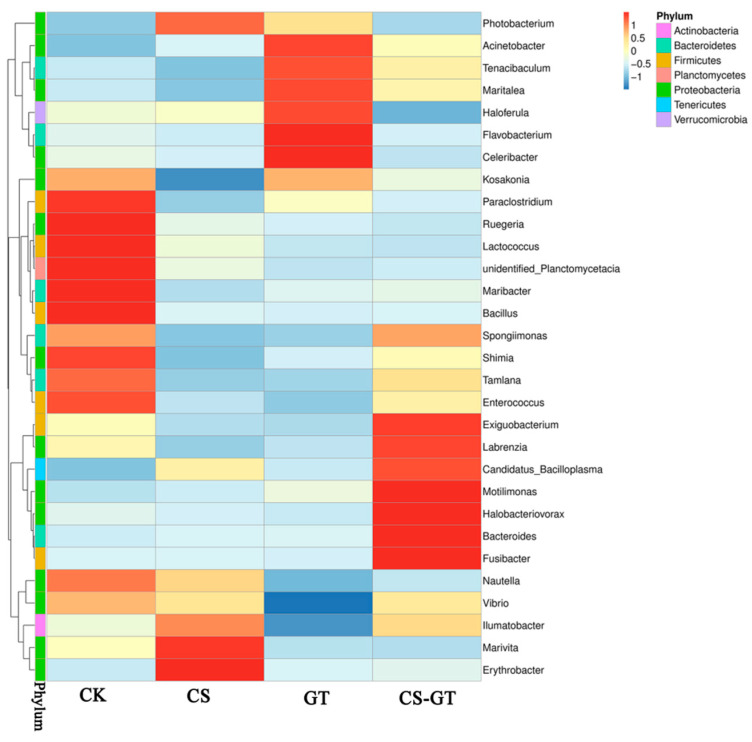
Heatmap analysis of the species abundance clustering in the top 30 at genus level in the gut (mean ± SD, n = 3).

The intestinal microbiota plays an important role in digestion, absorption, metabolism, and defense against pathogens [51]. In this study, the microbial compositions and changes in the gut in each group were analyzed. At the phylum level (Figure 11a), the bacteria were mostly Proteobacteria (37.37–88.87%), Firmicutes (4.11–79.88%), Tenericutes (2.09–16.75%), and Bacteroidetes (0.42–13.18%). Notably, Proteobacteria (78.34–88.87%) was most abundant in the CK and CS groups, while Firmicutes (51.82–79.88%) was most abundant in the CS-GT and GT groups. The higher abundance of Firmicutes in the CS-GT and GT groups could be associated with the presence of untreated fecal sewage [49]. The abundance of Proteobacteria was lower in the CS-GT and GT groups, which may be due to the suppression of *Vibrio* by GT and CS-GT. At the family level (Figure 11b), Mycoplasmataceae (10.04–81.02%), Rhodobacterales (21.22–41.91%), Enterococcaceae (15.74–22.46%), and Vibrionaceae (0.7–79.27%) were the main families in all groups. Enterococcaceae, which have been used as a probiotic for humans or animals (including shrimp) [52], and Rhodobacterales, which are also known to be potential probiotics for rainbow trout (*Oncorhynchus mykiss*) [53], were more abundant in the CS-GT and GT groups. This study suggested that CS-GT and GT may be able to alter the structure of the intestinal microflora and support the colonization of potential probiotics. Moreover, *Vibrio* is one of the most important diseases in shrimp aquaculture and causes serious mortality in shrimp worldwide [54]. *Vibrio* is widely distributed in marine environments and is typically among the most abundant flora in shrimp digestive systems [55,56]. Previous studies demonstrated that *Vibrio* is highly abundant in the guts of shrimps such as *Litopenaeus vannamei*, *Peneaus monodon*, and *Fenneropenaeus chinensis* [46]. As shown in Figure 12, in this study, *Vibrio* was predominant in the CK and CS groups, while being lower in the GT and CS-GT groups; this is because CS-GT and GT can inhibit the growth of Gram-negative bacteria [26]. 

#### 2.5.4. Microbial Function Prediction

**Figure 13:**
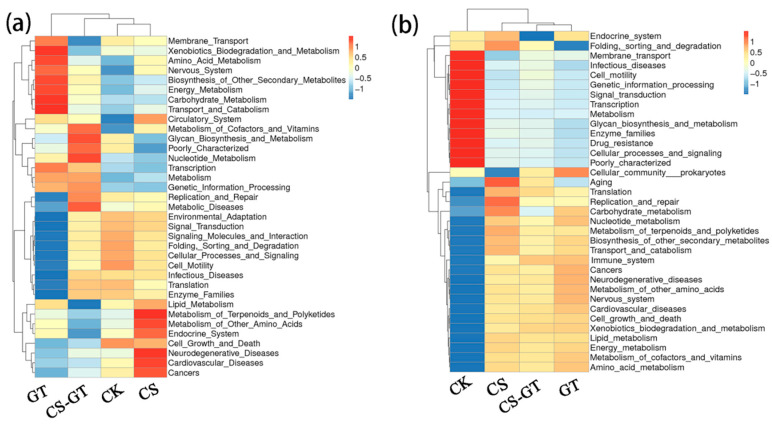
Heatmaps of microbial functions in the top 35 of level-2 for water (**a**) and gut (**b**) samples (mean ± SD, n = 3).

The microbial functions of the water and gut were predicted with PICRUSt2 (Phylogenetic Investigation of Communities by Reconstruction of Unobserved States) and Tax4Fun2 (an open-source R package that predicts the functional capabilities of microbial communities based on a 16S dataset) [57], respectively. The results showed that the water microbiota was enriched with functions related to glycan biosynthesis and metabolism, nucleotide metabolism, metabolism, genetic information processing, replication and repair, transcription, poorly characterization, and the metabolism of cofactors and vitamins (Figure 13a). The intestine microbiota was enriched with functions related to the immune system, amino acid metabolism, carbohydrate metabolism, translation, replication and repair, energy metabolism, the metabolism of cofactors and vitamins, xenobiotic biodegradation and metabolism, and lipid metabolism (Figure 13b). In particular, for the gut, the immune system is more abundant in the CS-GT group than in the CK group, and it is speculated that CS-GT can regulate the immune-related functions of shrimp.

#### 2.5.5. Correlation Analysis between Microbial Communities and Environmental Variables

**Figure 14:**
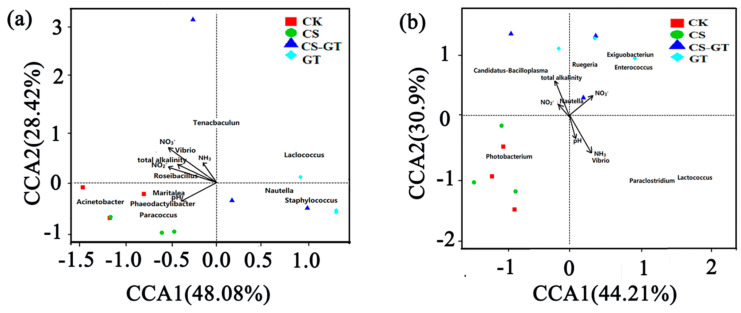
Canonical correlation analysis (CCA) ordination diagrams of environmental factors among water (**a**) and gut (**b**) samples (n = 3).

Canonical correlation analysis (CCA) was performed to determine the correlation between the physical–chemical constituents and microbial communities of the water and guts. For the water samples, five environmental variables explain 76.50% of the total change in microbial communities (Figure 14a). NO_3_^−^ had the greatest effect on the microbial community, followed by NO_2_^−^, pH, total alkalinity, and NH_3_; they were synergistic in their effects on the microbial structure. The five environmental variables were positively correlated with CK and CS, and negatively with CS-GT and GT; *Vibrio* was positively correlated with NH_3_, NO_3_^−^, and NO_2_^−^ concentrations, which were negatively correlated with GT and CS-GT dosage, suggesting that *Vibrio* was negatively correlated with GT and CS-GT dosage. It could be observed from Figure 14b that five environmental variables explained 75.11% of the total change in intestinal microbial community. NH_3_, NO_3_^−^, and total alkalinity had the greatest effects on the intestinal microbial community, followed by pH and NO_2_^−^. *Vibrio* was positively correlated with NH_3_ concentration, which was negatively correlated with GT and CS-GT dosage, suggesting that *Vibrio* was negatively correlated with GT and CS-GT dosage. The results show that CS-GT can regulate the abundances of *Vibrio* in water and intestines by adjusting the NO_2_^−^, NO_3_^−^, and NH_3_ concentrations.

## 3. Materials and Methods

### 3.1. CS-GT Synthesis

CS-GT was prepared according to our previous report with modifications [26]. Briefly, 3 g of CS (544 kDa, deacetylation degree: 95%) was firstly dissolved in 200 mL of acetic acid (1 v%) under magnetic stirring. A volume of 100 mL of sodium periodate solution (0.3 mol/L) was then added into the CS solution, which was allowed to react in the dark at 30 °C for 2 h. A 0.1 mol amount of ethylene glycol was then added to terminate the reaction. After dialysis and purification, the as-obtained oxidized chitosan was dried and collected for further modification. Prior to the graft, 2 g of oxidized chitosan was firstly dissolved in 100 mL of acetic acid solution (1 v%) and then mixed with 2 g of gentamicin sulfate under stirring for 4 h at 40 °C. Subsequently, the mixed solution was cooled to 30 °C, and its pH value was adjusted to 6.0. Then, 0.4 g of sodium cyanoborohydride (20% of oxidized chitosan) was added, and the reduction reaction was allowed to continue for 2 h at 30 °C. After dialysis and lyophilization, the CS-GT was stored in a desiccator for further use.

### 3.2. Experimental Design and Diets

The *Litopenaeus vannamei* (body weight: 6.05 ± 0.17 g) used in this study were obtained from East Island Marine Biology Research Base, College of Fisheries, Guangdong Ocean University (Zhanjiang, China). The shrimp were randomly allotted to twelve tanks (300 L, polyvinyl chloride polymer) at the rate of 30 shrimp tank^−1^. Prior to the experiment, all the shrimp were acclimated to the basal diet (commercial feed pellets twice daily at a feeding rate of 0.5% of shrimp bodies) and conditions (30 ± 0.5 °C, pH 7.8–8.2, and 32%_0_ salinity) for a week. 

For the experimentally induced infection implemented according to a previous study [58], the shrimp were exposed to *Vibrio parahaemolyticus* (10^6^ CFU ml^−1^) according to pre-experimental results. Subsequently, the supplements were coated onto the surface of commercial feed pellets using peanut oil, and the final supplement content in the experimental groups was designed as follows: control check group (i.e., CK group: no supplement), GT group (GT: 10 mg kg^−1^), CS group (CS: 250 mg kg^−1^), and CS-GT group (CS-GT: 50 mg kg^−1^). There were 40 shrimp tank^−1^ in each group. The residual food and feces were removed via a siphon, while the water in all the tanks was not renewed during the whole feeding trial.

### 3.3. Water Physicochemical Quality

Water samples were collected daily at 10 a.m. during the whole feeding trial. The pH and salinity were analyzed using pH and salinity meters, respectively. Total alkalinity was determined by analytical titration, while ammonia nitrogen (NH_3_), nitrite-nitrogen (NO_2_^−^), and nitrate-nitrogen (NO_3_^−^) concentrations were analyzed using the standard methods reported by Strickland and Parsons [59].

### 3.4. Hematological Profile

#### 3.4.1. Total Hemocyte Counts (THCs)

Initially, anticoagulant solution was prepared by dissolving 0.48 g of citrate, 1.32 g of sodium citrate, and 1.47 g of glucose in 100 mL of double distilled water, and then sterilizing the solution by autoclaving for further use. Six shrimp were randomly selected from each group during the feeding stage. Hemolymph (100 μL) was withdrawn from the ventral sinus of each shrimp using a 1 mL sterile syringe (25 gauge) containing 0.9 mL of anticoagulant solution. A drop of anticoagulant–hemolymph mixture (20 μL) above was transferred onto a hemocytometer for THC measurement using an inverted phase-contrast microscope (Leica DMIL, Munich, Germany). Each sample was repeated in triplicate, and the average was recorded. 

#### 3.4.2. Hemocyte Apoptosis Analysis 

Hemocyte apoptosis assays were conducted with an Annexin V-FITC/PI Apoptosis Detection Kit (Beyotime, Shanghai, China) at Day 3 according to the manufacturer’s protocol. The hemolymph was obtained using similar procedures with the final volume fraction of the anticoagulant solution improved to 50%. The mixture was then centrifuged at 1000 rpm at 4 °C for 5 min to obtain the hemocyte microspheres, which were resuspended in PBS (pH 7.4) and adjusted to a cell density of 1 × 10^6^ cells/mL for another centrifugation using the same parameters. The final hemocyte microspheres obtained were stained with Annexin V-FITC and PI according to the manufacturer’s instructions. The percentage of apoptotic hemocytes was determined by flow cytometry (BDFACSC anto II, Franklin Lake, NJ, USA) in triplicate.

### 3.5. Nonspecific Immunity Parameters 

Six shrimp (body weight: 6.05 ± 0.17 g) were randomly selected from each group at pre-determined time intervals (Days 0, 1, 2, and 3) and dissected, with their hepatopancreases immediately stored in liquid nitrogen for antioxidant and immune enzyme activity analysis. Prior to analysis, hepatopancreas samples were homogenized in ice-cold phosphate buffer (1:10 dilution) and then centrifuged for 20 min (4 °C, 3000 rpm) to obtain the supernatant for further use. 

Superoxide dismutase (SOD), glutathione peroxidase (GSH-PX) and glutathione (GSH), lysozyme (LSZ), acid phosphatase (ACP), alkaline phosphatase (ALP), and phenoloxidase (PO) were measured using commercial detection kits (Jianglai Bioengineering Institute, Shanghai, China) according to the manufacturer’s protocols.

### 3.6. Intestinal Histology

Three shrimp were randomly selected from each tank of the four groups at Day 3 and dissected, with the mid-gut immediately stored in Bouin’s solution for later use. After fixation in Bouin’s solution and dehydration in a gradient of ethanol solutions, the mid-gut segments were cleaned in toluene and embedded in paraffin. Cross sections (around 5 µm) of the embedded samples were cut using a rotary microtome and then stained with hematoxylin and eosin (H&E), and imaged with a light microscope (Olympus, Nikon, Tokyo, Japan).

### 3.7. Microbiota Analysis

#### 3.7.1. DNA Extraction

For DNA extraction, approximately 1 L of rearing water (in triplicate) was filtered through a nylon mesh (100 µm pore size) and a polycarbonate membrane (0.22 µm pore size) on Day 3, respectively. The membranes obtained were stored at −80 °C for further genomic DNA extraction and analysis. Prior to gut sampling, six shrimp (in triplicate) were rinsed twice using 75 v% ethanol, and their gastrointestinal tracts were excised using a sterile toothpick. The gut tissue obtained was then transferred into a sterilized EP tube and immediately stored in liquid nitrogen for genomic DNA extraction and analysis.

Total genomic DNA was extracted using the CTAB/SDS method. DNA concentration and purity were assessed on 1% agarose gels. According to the concentration, DNA was diluted to 1 ng/μL using sterile water.

#### 3.7.2. 16S rRNA Gene Sequencing

In order to determine the microbial communities of the guts and rearing water, the amplification and sequencing of the V4 region of the bacterial 16S rDNA gene was conducted in triplicate using 515 F and 806 R as primer sets. 

All the PCR reactions were carried out in 30 μL reactions: 15 μL of Phusion^®^ High-Fidelity PCR Master Mix (New England Biolabs, Massachusetts, MA, USA), 2 μM forward and reverse primers, 10 μL of template DNA (5–10 ng), and 2 μL of H_2_O. Thermal cycling consisted of an initial denaturation at 98 °C for 1 min, followed by 30 cycles of denaturation at 98 °C for 10 s, annealing at 50 °C for 30 s, elongation at 72 °C for 30 s, and, finally, 72 °C for 5 min. After amplification, the same volume of 1 × TAE loading buffer (contained SYB green) was mixed with PCR products, and electrophoresis was performed on a 2% agarose gel for detection. Samples with a bright main strip between 400 and 450 bp were chosen for further experiments. The PCR products were mixed in equidensitic ratios. Then, the mixture was purified with a Gene JET Gel Extraction Kit (Thermo Scientific, Waltham, MA, USA).

Sequencing libraries were generated using an NEB Next^®^ Ultra™ DNA Library Prep Kit for Illumina (New England Biolabs, Massachusetts, MA, USA) following the manufacturer’s recommendations, and index codes were added. The library quality was assessed on the Qubit@ 2.0 Fluorometer (Thermo Scientific, Waltham, MA, USA) and Agilent Bioanalyzer 2100 system. Finally, the library was sequenced on an Illumina HiSeq platform (Novogene, Beijing, China) and 250 bp paired-end reads were generated.

#### 3.7.3. Sequencing Data Analysis

Paired-end reads from the original DNA fragments were merged by using a very fast and accurate analysis tool, Fast Length Adjustment of Short reads (FLASH-1.2.11,McKusick-Nathans Institute of Genetic Medicine, Johns Hopkins University School of Medicine, Baltimore, MD, USA), which is designed to merge paired-end reads in the case of overlap between reads1 and reads2. Paired-end reads were assigned to each sample according to the unique barcodes. Sequences were analyzed using the Quantitative Insights Into Microbial Ecology software (QIIME2, Department of Chemistry and Biochemistry, University of Colorado, Boulder, CO, USA).

Alpha (within samples) and beta (among samples) diversities were analyzed based on in-house Perl scripts in QIIME2. The reads were filtered with QIIME2 and clustered into operational taxonomic units (OTUs) at an identity threshold of 97%. A Venn diagram was generated to identify unique and shared OTUs. Alpha diversity indices including Chao1, Shannon, Simpson, and abundance-based coverage estimator (ACE) were applied to analyze the complexity of species diversity. Chao1 and ACE were used to assess community richness, whereas Shannon and Simpson were used to assess community diversity. The different taxonomic levels were transformed to relative abundance counts to draw bar plots. In order to investigate the similarity of the community structure among different samples, principal coordinates analysis (PCoA) was performed using weighted-uniFrac distances, respectively. PICRUSt2 (Department of Microbiology and Immunology, Dalhousie University, Halifax, NS, Canada) is a biological information software package for predicting metagenome functions based on Marker genes (such as 16S rRNA). Tax4Fun2 (Evolution and Ecology Research Centre, School of Biological, Earth and Environmental Sciences, University of New South Wales, NSW, Australia) is an R program package for function prediction for intestinal, soil, and other environmental samples based on a 16S Silva database [57]. Canonical correspondence analysis (CCA) based on a unimodal model was used to address the relationship between microbial communities and water quality indicators.

### 3.8. Statistical Analysis

All data, presented as mean ± SD, were subjected to a one-way analysis of variance (ANOVA) using Statistical Package for the Social Sciences (IBM SPSS v20.0, Inc., 2010, Chicago, IL, USA), followed by the testing of mean differences using Tukey’s multiple comparison tests. The value of *p* < 0.05 was set for statistical significance.

## 4. Conclusions

Our results show that the total hemocyte counts, antioxidant activities, immune enzyme activities, and intestinal morphology of *Litopenaeus vannamei* were improved after dietary supplementation with a chitosan–gentamicin conjugate. Meanwhile, the abundances of *Vibrio* in the water and guts of *Litopenaeus vannamei* were significantly reduced after dietary supplementation with the chitosan–gentamicin conjugate. This study provides a new research foundation and ideas for the future development of chitosan conjugates in aquaculture.

## Figures and Tables

**Table 1:** Alpha diversity indices of microbiota in water and gut.

Samples	Group	Richness Estimate		Diversity Estimate		
		Chao1	ACE	Shannon	Simpson	Good’s Coverage
water	CK	475.84 ± 25.31 ^a^	478.52 ± 45.45 ^a^	5.27 ± 0.27 ^a^	0.79 ± 0.01 ^a^	0.9995 ± 0.0018 ^a^
	CS	461.95 ± 15.54 ^a^	432.83 ± 44.36 ^a^	5.19 + 0.14 ^a^	0.83 ± 0.02 ^a^	0.9995 ± 0.0004 ^a^
	CS-GT	435.39 ± 91.77 ^a^	411.55 ± 75.57 ^a^	4.72 ± 0.57 ^a^	0.88 ± 0.04 ^b^	0.9995 ± 0.0004 ^a^
	GT	417.15 ± 5.68 ^a^	404.70 ± 10.55 ^a^	4.17 ± 0.22 ^b^	0.92 ± 0.03 ^b^	0.9995 ± 0.0002 ^a^
gut	CK	346.18 ± 90.64 ^a^	348.52 ± 88.57 ^a^	4.51 ± 2.57 ^a^	0.78 ± 0.14 ^a^	0.9997 ± 0.0007 ^a^
	CS	378.42 ± 98.21 ^a^	382.83 ± 96.31 ^a^	4.81 ± 0.32 ^a^	0.72 ± 0.03 ^a^	0.9997 ± 0.0003 ^a^
	CS-GT	324.53 ± 0.53 ^a^	339.37 ± 13.50 ^a^	4.63 ± 0.56 ^a^	0.81 ± 0.05 ^a^	0.9997 ± 0.0005 ^a^
	GT	287.03 ± 23.05 ^a^	291.53 ± 21.06 ^a^	4.18 ± 1.33 ^a^	0.88 ± 0.17 ^a^	0.9997 ± 0.0004 ^a^

Values are expressed as mean ± SD (n = 3), and values with significant differences are labeled with different letters within the same row, a: *p* > 0.05, b: *p* < 0.05.
